# Aromatic L‐amino acid decarboxylase deficiency in 17 Mainland China patients: Clinical phenotype, molecular spectrum, and therapy overview

**DOI:** 10.1002/mgg3.1143

**Published:** 2020-01-23

**Authors:** Weiqian Dai, Deyun Lu, Xuefan Gu, Yongguo Yu, the Mainland Chinese League of AADC Rare Disease

**Affiliations:** ^1^ Department of Pediatric Endocrinology and Genetic Metabolism Shanghai Institute for Pediatric Research Xinhua Hospital School of Medicine Shanghai Jiao Tong University Shanghai China

**Keywords:** Aromatic L‐amino acid decarboxylase (AADC）deficiency, Mainland China, genotype‐enzyme‐clinical phenotype correlation

## Abstract

**Background:**

Aromatic L‐amino acid decarboxylase deficiency (AADCD) is a rare, autosomal recessive inherited disorder which is characterized by neurological and vegetative symptoms. To date, only 130 patients with AADCD have been reported worldwide.

**Methods:**

We demonstrated 14 previously undescribed patients together with three reportedly patients in Mainland China. Full clinical information was collected, and disease‐causing variants in the DDC gene were detected.

**Results:**

The common clinical manifestation of patients, including intermittent oculogyric crises, retarded movement development, and autonomic symptoms. Notably, a patient showed bone‐density loss which have not been reported and two mildly phenotype patients improved psychomotor function after being prescribed medication. The most common genotype of Mainland Chinese AADCD is the splice‐site variant (IVS6+4A> T; c.714+4A> T), which accounts for 58.8%, followed by c.1234C>T variant. Three novel compound heterozygous variants, c. 565G>T, c.170T>C, and c.1021+1G>A, were firstly reported. It is important to recognize the milder phenotypes of the disease as these patients might respond well to therapy. Besides, we discovered that patients may presented with milder if found to be compound heterozygote or homozygote for one of the following variants c.478C>G, c.853C>T, c.1123C>T, c.387G>A, and c.665T>C.

**Discussion:**

The clinical data of the cohort of 17 patients in Mainland China broaden the clinical, molecular, and treatment spectrum of aromatic L‐amino acid decarboxylase deficiency.

## Graphical abstract

The most intriguing aspects of our study is that we present clinical details on a cohort of 17 patients in Mainland China with broad clinical variability, three novel variants in DDC gene, and different responses to treatment. In addition, we explored *DDC* genotype with mild or moderate clinical phenotype correlation. In short, our study expanded the clinical spectrum of AADCD and contributes to the knowledge of the genotype and phenotype correlation for the DDC gene.

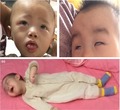

## 1 INTRODUCTION

Aromatic L‐amino acid decarboxylase (AADC) deficiency (AADCD; OMIM® #608643) is an extremely rare, autosomal recessive inherited disorder that is caused by pathogenic variants in the DDC gene, which is located on chromosome 7p12.2‐p12.1, and is characterized by neurological and vegetative symptoms that usually begin during infancy or childhood. Aromatic L‐amino acid decarboxylase, encoded by the DDC gene, catalyzes the decarboxylation step of the monoamine neurotransmitter biosynthetic pathway, using pyridoxal‐5′‐phosphate as a cofactor, in which 5‐hydroxytryptophan and levodopa (L‐dopa) are irreversibly converted to serotonin and dopamine, respectively (Allen, Land, & Heales, 2009; Pons et al., 2004). In this biogenic amine synthetic pathway, catecholamines and melatonin are downstream of dopamine and serotonin, respectively. As a consequence of AADC deficiency, levels of biogenic amines, including dopamine, norepinephrine, epinephrine, and serotonin, are reduced, and the levels of biogenic amine precursors containing L‐dopa and 5‐hydroxytryptophan are increased. When dopamine and noradrenaline are absent, characteristic clinical manifestations, including oculogyric crises, hypokinesia, dystonia, ptosis, and autonomic dysfunction, are observed. Other clinical features, such as sleep disorders, mental problems, and gastrointestinal dysfunction, are also observed as a result of serotonin deficiency. The genetic and clinical spectrum of AADCD are heterogeneous and vary extensively among patients. A diagnosis is usually made based on a characteristic cerebrospinal fluid neurotransmitter metabolite profile and low or absent plasma AADC enzyme activity in conjunction with the identification of pathological variants in the DDC gene (Wassenberg et al., 2017). To date, more than 100 cases were identified worldwide. Notably, AADCD is more prevalent in Southeast Asia, especially in Taiwan and Japan, due to an underlying founder effect. However, up to now, very few patients have been reported in Mainland China. Here, we report 14 previously undescribed patients (mean age, 2.37 years) and three previously reported Mainland Chinese patients with AADCD (Dai, Ding, & Fang, 2019; Zhu & Yu, 2017). In this study, we focused on clinical manifestations, drug treatment, and molecular studies of the index subjects, with an aim to expand the molecular and phenotypic spectrum of AADCD.

## 2 METHODS

### 2.1 Patients

The study was approved by Ethics Committee of Xinhua Hospital, School of Medicine, Shanghai Jiao Tong University (XHEC‐C‐D‐2019‐041) and informed parental consent was obtained before including the patients in the study. From 2017 to 2019, we recruited 14 previously unreported patients who were diagnosed with AADCD based on characteristic clinical manifestations suggestive of AADCD and identification of DDC gene mutations in Mainland China from the China League of AADC Rare Disease. Three previously reportedly patients, two siblings and a boy from Mainland China, were also discussed in our study (Dai et al., 2019; Zhu & Yu, 2017). Clinical information was collected from patients’ medical history and personal information of treating physicians using a standardized evaluation protocol.

### 2.2 Molecular analysis of the DDC gene and AADC protein

Genomic DNA was extracted from patient peripheral blood samples. Disease‐causing mutations in the DDC gene were detected by high‐throughput sequencing, and the results were confirmed by Sanger sequencing. The raw data of gene sequencing were collected, and it have been re‐analyzed and re‐confirmed by our research team. To gain insight into the effects of the detected *DDC* mutations, the three‐dimensional structures of the mutant AADC proteins were visualized via comparative protein modeling by PyMol.

## 3 RESULTS

### 3.1 Clinical findings

**Figure 1:**
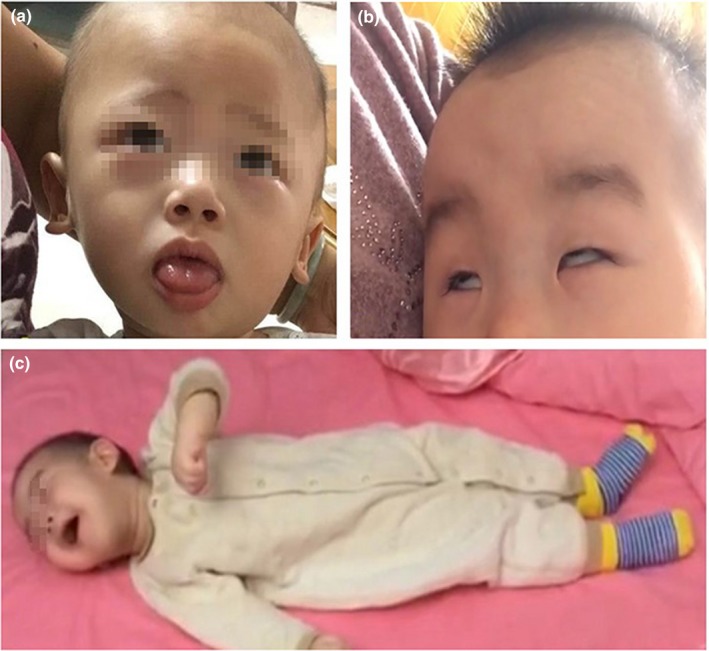
Characteristic clinical manifestations of patients with aromatic L‐amino acid decarboxylase deficiency in Mainland China. (a) Representative photograph of common clinical phenotype only in Mainland China, involuntary tongue thrusting, in a 1‐year‐old boy. (b) The index subject is a 11‐month‐old boy, who presented with involuntary quick movements of the eyeball in a horizontal direction or upward deviation of the eyes (oculogyric crisis). (c) In this picture, an 8‐month‐old patient in an episode of mild opisthotonos is shown, in which the back arches, the head bends back, and the heels flex toward the back

In our cohort, the onset of pathological symptoms in AADCD typically occurs between 2 and 5 months of age. At the first visit, parents of patinets mainly complained of poor head control and occasionally upward deviation of the eyes. However, all of them subsequently demonstrated the characteristic clinical hallmarks, including intermittent oculogyric crises (Figure 1b), comprehensive developmental retardation, and dystonia. Intriguingly, for all untreated patients, the oculogyric crises not only persisted for 6–8 hr twice a week but also peaked in the evening at around 6 o'clock. In addition, all the patients uniformly reported that the oculogyric crises worsened at about 10–12 months of age. Patients were never able to control their head, sit, and crawl independently or with assistance. Besides, during an acute episode of disease, part patients presented with the typical symptoms of mild opisthotonos (Figure 1c).

In addition to these characteristic neurological symptoms, all patients also presented with heterogeneous autonomic symptoms and mental problems (Table 1). It is interesting that two common manifestations were observed in our cohort which are classical findings for many inherited metabolic disorders. Firstly, small hands and feet were observed in 85.7% of cases; in contrast, nearly all Taiwanese AADCD patients have small hands and feet (Lee, Tsai, Chi, Chang, & Lee, 2009). It was not altogether surprising, as likely as not, the classical clinical features possess the characteristic of ethnic. With regard to another common phenotype, most patients presented with unconscious tongue thrusting (Figure 1a), which resembles that we can observed in 21 trisomy syndromes. Notably, a patient presented with bone bone‐density loss which Z‐score is −3 which have not been reported in literature.

**Table 1:** Clinical findings in 14 previously unreported patients harboring *DDC* variants

Patient no. gender age at onset current age	1 F 5 m 1 year 3 months	2 M 4 m 3 years 10 months	3 F 3 m 1 year 3 months	4 F 3 m 4 years 2 months	5 M 3 m 1 year	6 F 4 m 3 years 8 month	7 F 3 m 4 years^*^	8 M 3 m 2 years 5 months	9 M 4 m 1 year 8 months	10 F 4 m 1 year 9 months	11 F 2 m 5 years	12 F 4 m 1 year 5 months	13 F 3 m 2 years 8 months	14 M 3 m 1 year 4 months	No. of patients with the phenotype
Movement disorders															
Oculogyric crises	+	+	+	+	+	+	+	+	+	+	+	+	+	+	14/14
Dystonia	+	+	+	+	+	+	+	+	+	+	+	+	+	+	14/14
Hypokinesia	+	+	+	+	+	+	+	+	+	+	+	+	+	+	14/14
Poor head control	+	+	+	+	+	+	+	+	+	+	+	−	−	+	12/14
Athetosis	−	−	+	−	−	+	−	−	−	−	−	+	−	−	3/14
Myoclonus/ Tremor	−	+	+	+	−	−	−	−	−	−	+	+	−	+	6/14
Developmental delay	+	+	+	+	+	+	+	+	+	+	+	+	+	+	14/14
Mental problems															
Increased startle	+	+	+	+	+	+	+	+	+	+	+	+	+	+	14/14
Irritability	+	+	+	+	+	−	+	+	+	+	−	+	−	−	10/14
Dysphoria	+	+	+	+	+	−	+	+	+	+	−	−	−	+	10/14
Excessive crying	−	+	+	+	+	−	+	−	−	+	−	+	−	+	8/14
Autonomic nervous system															
Hyperhidrosis	+	+	+	+	+	−	+	+	+	+	+	+	+	+	13/14
Ptosis	−	+	+	+	+	+	+	+	+	+	+	+	+	−	12/14
Excessive drooling	+	+	+	+	+	+	+	+	−	+	+	+	−	+	12/14
Nasal congestion	+	+	+	+	+	+	−	+	+	−	−	−	+	+	10/14
Miosis	−	−	+	−	+	+	−	−	−	−	−	−	+	−	4/14
Obstipation	+	−	+	−	−	−	−	−	−	+	−	−	−	+	4/14
Diarrhea	−	−	−	−	−	−	−	−	−	−	−	−	+	−	1/14
Temperature instability	−	+	−	+	+	−	−	−	−	−	−	−	−	+	3/14
Cardiovascular	−	−	−	−	−	−	−	−	−	−	−	−	−	−	0/14
Other															
Failure to thrive	+	+	+	+	+	+	+	+	−	+	+	+	+	+	13/14
Small hand and feet	+	+	−	+	+	+	+	+	+	+	+	+	+	−	12/14
Tongue thrusting	+	+	+	+	+	+	+	+	+	+	−	+	−	+	12/14
Fatiguability	+	+	+	+	+	+	+	+	−	−	+	+	+	+	12/14
Dysarthria	+	+	+	+	+	+	+	+	+	+	+	−	−	+	12/14
Insomnia	−	+	+	+	+	+	+	+	+	−	+	+	−	+	11/14
Feeding problems	−	+	−	+	+	+	+	+	+	+	−	−	−	−	8/14
Gastrointestinal reflux	−	+	−	+	+	−	+	+	−	+	−	+	−	−	7/14
Epileptic seizures	−	−	+	−	−	+	−	−	+	+	+	−	−	−	5/14
Hypoglycemia	−	−	−	−	−	−	−	+	−	−	−	−	−	+	2/14
Hyperprolactinemia	−	−	−	−	−	−	−	+	−	−	−	−	−	+	2/14
Bone‐density loss	−	−	−	−	−	−	−	−	−	−	−	+	−	−	1/14
Accessory examinations															
MRI†, *	*N*	1	2	2	*N*	3	*N*	2 + 4	*N*	*	*	5	2	*N*	
EEG	*N*	A	A	A	*N*	A	A	*N*	*N*	A	A	*N*	*N*	*N*	
Organic acids profile	*N*	*N*	*N*	*	*N*	*N*	*N*	*N*	*	*	*N*	*N*	*	*N*	
3‐OMD from dried blood spot	*	A	*	*	*	*	A	A	A	*	*	*	*	*	
Disease phenotype	Severe	Severe	Severe	Severe	Severe	Severe	Severe	Severe	Severe	Severe	Severe	Moderate	Moderate	Severe	

Note

Disease phenotypes: severe ‐ profound hypotonia completely unable to control head, developmental delay, and frequently oculogyric crises; mild ‐ mild developmental delay, ambulatory without assistance, and mild intellectual disability; and moderate ‐ in between severe and mild.

M, male; F, female. In clinical phenotype, the symbol of “+” means the patient presented with the corresponding clinical phenotype;the symbol of “−” means the patient did not presented the clinical phenotype. In accessory examinations, the symbol of “N” means the results of corresponding accessory examination were normal; the symbol of “A” means the results were abnormal or nonspecific; the symbol of “*” means there are no available data.

† In MRI findings, the number 1 means reduced brain volume; 2 means increased extracerebral space; 3 means enlargement of the subarachnoid spaces; 4 means degenerative changes in the white matter; and 5 means hypomyelination.

Heterogeneous structural brain abnormalities were observed in 7 of 12 patients who had undergone MRI, including increased extracerebral space in the bilateral frontotemporal regions (four cases), degenerative changes in the white matter (one case), hypomyelination (one case), and reduced brain volume or enlargement of the subarachnoid spaces (one case). EEG results were normal in seven cases (50%), nonspecific in five cases (35.7%), and abnormal in two cases (14.28%), with generalized slow background, spike waves, sharp waves, and spike (or sharp) slow wave complexes. The organic acid profiles in urine and blood were normal in all tested patients. Four index patients screened in Taiwan presented with elevated 3‐O‐methyldopa (3‐OMD) levels in dried blood spots screen. A complete list of clinical phenotype and auxiliary examination results are given in Table 1.

As soon as AADCD was confirmed, all index patients were treated with drug. All index patients were treated with pyridoxine which function as an enzyme cofactor and can bolster the catalytic activity of the remaining enzyme. Dopamine agonists which mimic the dopamine function under physiological condition by directly activating postsynaptic dopamine receptors were used as well, including bromocriptine (one case), pergolide (five cases), and the rotigotine patch (four cases). Besides, eight patients were treated with MAO inhibitors blocking the breakdown of dopamine and serotonin, thus increasing monoamine level in brain. The responses are not satisfactory particularly in patients having severe symptoms even when drugs were administered in combination. It is worth to mentioning that two patients improved psychomotor function after being medication. Patient 12 improved significantly after selegiline and L‐dopa treatment. She started to babble at 1 year and 5 months, and her episodes of oculogyric crisis became primarily restricted to periods of fatigue. Another patient, patient13, responded dramatically to bromocriptine, and she can able to eat food with her fingers when she turned 2 years old.

### 3.2 Molecular studies

Fourteen different variants in the DDC gene were identified in the 17 patients. For the prediction of variant severity, the web‐based tools SIFT (https://sift.bii.a-star.edu.sg/), PolyPhen‐2 (http://genetics.bwh.harvard.edu/pph2/), and MutationTaster2 (http://www.mutationtaster.org/) were used. All identified *DDC* variants were predicted as possibly damaging, probably damaging, or deleterious (Table 2). The homozygous IVS6+4A>T variant alone was detected in three patients, and it also was detected as a compound heterozygous variant in another 41.18% (7/17) of the patients, making it the common variant, followed by the compound heterozygous variant c.1234C>T (p.R412W) (17.6%). In addition, three compound heterozygous variants, c.565G>T (p.D189Y), c.170T>C (p.I57T), and c.1021+1G>A, were firstly described in our research (ClinVar, OMIM, and GWAS catalog).

**Table 2:** Summary of *DDC* gene mutations and the prediction results by the web‐based tools in our study

Patient	Mutation	AA‐change	Exon	Variation type	Protein domain	PROVEAN score	PolyPhen core	SIFT score	Mutation taster score
1、2、14	714+4A>T		6	Splicing	Large domain				
				
3、4	714+4A>T		6	Splicing	Large domain				
	1234C>T	R412W	13	Missense	C‐domain	Deleterious	Probably damaging	Deleterious	101
5、6	714+4A>T		6	Splicing	Large domain				
	1297dupA	I433N	14	Missense	C‐domain	Deleterious	Probably damaging	Deleterious	
7、8	714+4A>T		6	Splicing	Large domain				
	106G>A	G36R	2	Missense	*N*‐domain	Deleterious	Probably damaging	Deleterious	125
9	714+4A>T		6	splicing	Large domain				
	175G > A	D59N	2	Missense	*N*‐domain	Deleterious	Probably damaging	Deleterious	23
10	179T>C	V60A	2	Missense	*N*‐domain	Deleterious	Possibly damaging	Deleterious	64
	1234C>T	R412W	13	Missense	C‐domain	Deleterious	Probably damaging	Deleterious	101
11	170T>C	I57T	2	Missense	*N*‐domain	Neutral	possibly damaging	Deleterious	89
	1234C>T	R412W	13	Missense	C‐domain	Deleterious	Probably damaging	Deleterious	101
12	478C>G	R160G	5	Missense	Large domain	Deleterious	Probably damaging	Deleterious	125
	565G>T	D189Y	5	Missense	Large domain	Deleterious	Probably damaging	Deleterious	160
13	1,021+1G>A		9	Splicing					
	299G > C	C100S	3	Missense	Loop2	Deleterious	Possibly damaging	Deleterious	112
15†, *	1063dupA	I355fs	10		loop3				
	250A>C	S84R	2	Missense	loop1	Neutral	Possibly damaging	Deleterious	110
16/17†, *	1040G>A	R347Q	10	Missense	loop3	Deleterious	Possibly damaging	Deleterious	

Note

Prediction:(1) PROVEAN prediction: Default threshold is −2.5, that is variants with a score equal to or below −2.5 are considered "deleterious," whereas variants with a score above −2.5 are considered "neutral.” (2) PolyPhen‐2 prediction: Probably damaging with a score of 1, in contrast, possibly damaging with a score under 1. (3) SIFT prediction: Amino acids with probabilities < 0.05 are predicted to be deleterious, whereas variants with a score above 0.05 are considered "neutral.” (4) Mutation taster prediction: Scores range from 0.0 to 215. The more they score, the more deleterious protein mutations.

* Patients 14 have been reported by Lifang Dai, whereas patients15/16 by Jie Zhu.

**Figure 2:**
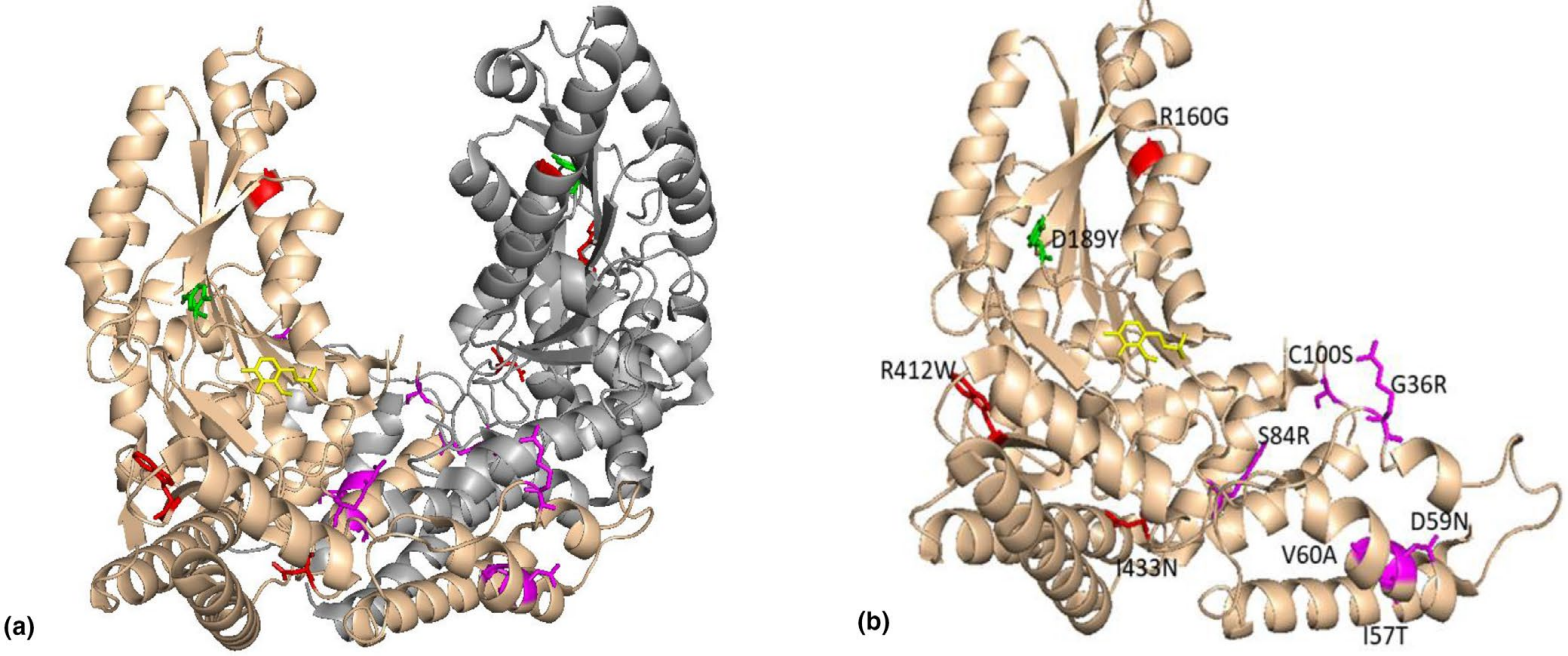
We used the crystal structure of AADC molecule from Sus scrofa. The structure of AADC comprises a tightly associated dimer with two catalytic pockets lining the dimer interface, and the active site of the enzyme is located near the monomer‒monomer interface. The left panel (a) shows the dimeric AADC molecule in which the two chains are represented as tan and grey ribbons. Mutated amino acids are shown as follows: near the active site (green), at the dimer interface (magenta), and in the peripheral region (red). PLP molecules are also shown in yellow. The right panel (b) of the figure shows stick representations of the side chains of the AADC molecule. We used same colors to mark the different mutant amino acids in the left and right panels of the figure

The crystal structure of human AADC in the holo form is not available; however, the protein from *Sus scrofa* (PDB ID: http://www.rcsb.org/pdb/search/structidSearch.do?structureId=1JS3), which shares 88% sequence identity with the human enzyme (Burkhard, Dominici, Borri‐Voltattorni, Jansonius, & Malashkevich, 2001), is available, thus it was used as a model for human AADC. We used PyMol to visualize the crystal structure of AADC (MMDB ID: 94,465) and explore the pathogenicity of these gene changes. DDC molecule contain three loops, including loops 1 (residues 66–84), 2 (residues 100–110), and 3 (residues 323–357), that play a pivotal role in the switch between the apo and holo forms of the enzyme (Montioli et al., 2014). As shown in Figure 2, the 10 identified *DDC* missense mutations were not evenly distributed over the AADC dimer. The identified *DDC* mutants can be categorized into two groups, one group that affects apo‒holo conversion and the other group that is close to the catalytic site. Three mutants that mapped to loops 1 (S84R), 2 (C100S), and 3 (R347Q), affected the proper apo‒holo transition, thus decreasing the catalytic efficiency. Moreover, R347Q is not only located at a central position in loop 3 at the entrance to the active site cavity, but it is also close to the decarboxylase catalytic element (residues 328–339). With respect to the other detected mutations, G36R, I57T, D59N, and V60A are located at the dimer interface; R412W and I433N are located in peripheral regions, and R160W and D189Y are located in the large domain near the active site.

**Figure 3:**
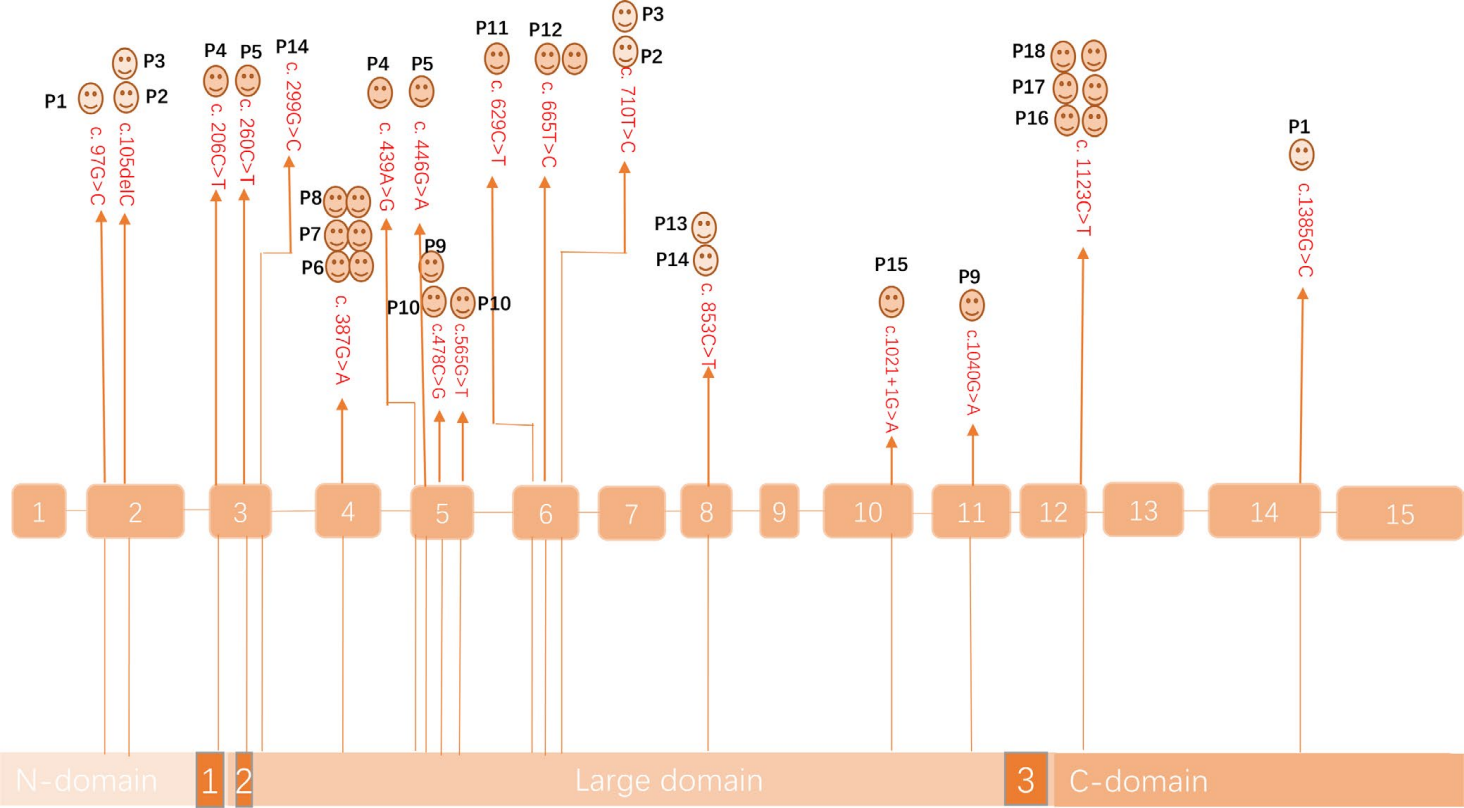
Schematic diagram of DDC gene and aromatic L‐amino acid decarboxylase. The upper rectangles represent the exons, and the lower rectangles represent the protein domains. The human DDC gene consists of 15 exons spanning more than 85 kilobases. The overall structure of the protein is a tightly associated dimer in which each monomer consists of a N‐terminal domain (residues 1–85), large domain (residues 86–360, with the PLP binding cleft and core domain), and C‐domain (residues 361–475). The smiley faces represent the patients, and the darkness of the color indicates the severity of their disease phenotypes, with the darkest color indicating the most severe phenotypes, and the lightest color indicating milder phenotypes. P10 and P15, who presented with moderate phenotype, are index subjects of our cohort. Besides, the other patients who have been reported in literature, showed mild or moderate phenotypes

We firstly explore *DDC * genotype with mild or moderate clinical phenotype correlation. As shown in the DDC gene exon structure schematic diagram, an interesting phenomenon that identified *DDC* variants in literature associated with milder phenotype obviously aggregated in exon5‐exon6 (436‐714) residues can be found (Figure 3). Moreover, it seems that patients presented with mild or moderate if found to be compound heterozygote or homozygote for one of the following missense or nonsense mutations: c.478C>G, c.853C>T, c.1123C>T, c.387G>A, c.665T>C (Figure 3).

We paid attention on the position of the residues Arg160, Asp189, and Cys100 in AADC molecule as a result of they may cause milder phenotypes. The Arg160 residue is located at the interface of the two subunits. The side chain of Arg160 not only establishes intermolecular hydrogen bonds with the main chain carbonyl groups of Leu200 and Ile201 but also is involved in salt‐bridge interactions with Glu181 and Glu196. The analysis suggests that the R160W substitution causes a total loss of the Arg160‐Leu200/Ile201 interactions, and loss of Arg160 disrupts the salt‐bridge interaction with Glu181 and Glu196, inducing local destabilization of the dimer structure. In addition, the previously literature reported that the R160W substitution affects both the coenzyme binding affinity and the catalytic efficiency of the DDC (Montioli, Janson, Paiardini, Bertoldi, & Borri Voltattorni, 2018). Therefore, R160W likely alter protein dimerization and change the corresponding kinetic characteristics for the L‐dopa substrate. The Asp189 residue is near the active site of the enzyme, but the side chain points toward the outside of the enzyme structure and may not form hydrogen bonds or salt bridges with adjacent important amino acid residues. The loss of Asp189 may only induce slightly local destabilization of the dimer structure. Cys100 is mapped to loop 2 and is involved in a significant open‐closed conformational change. The side chain of Cys100 is pointed toward the outside of the AADC structure and is adjacent to Ile101 and Phe103 from the neighboring subunit, which are located in the L‐dopa binding site. Cys100 also faces Thr82, which is involved in substrate binding and cofactor stabilization. The C100S substitution may mainly affect proper switching between the apo and holo forms of the enzyme and indirectly disrupt L‐dopa and cofactor binding.

## 4 DISCUSSION

It is known that there is an increased prevalence of AADCD in southern Chinese descent. Over the past few years, about 70 patients diagnosed with AADCD have been reported in Taiwan (Hwu, Chien, Lee, & Li, 2018; Lee et al., 2009); however, only three cases have been reported in Mainland China. To make up for the gap, our research team recruited 14 previously unreported patients form Mainland China including North and South China. Moreover, a thorough investigation of the patients’ family trees showed no evidence of Taiwanese heritage.

The key symptoms of AADCD are hypotonia, movement disorders, developmental delay, and autonomic symptoms (Brun et al., 2010). Intermittent oculogyric crises, retarded movement development, dystonia and autonomic symptoms were common clinical signs in our patients, which is compatible with previous reports (Wassenberg et al., 2017). Developmental delays, poor head control, and intermittent oculogyric crises, are the most common complaints at onset. Thus, if a child presents with oculogyric crises accompanied by unexplained movement and developmental delays, especially dystonia, AADCD should be considered. Four patients presented epileptic seizures with EEG findings, but only nine patients with epileptic seizures have been reported worldwide (Brun et al., 2010; Chang, Sharma, & Marsh, 2004; Helman, Pappa, & Pearl, 2014; Ito et al., 2008; Manegold et al., 2009). Although epileptic seizures are uncommon in AADCD, the differentiation of epileptic seizures from involuntary nonepileptic movements, is indispensable for the appropriate antiepileptic drugs treatment of patients. Patient 12 presented with bone‐density loss which has not been previously reported. Since catecholamines are known to be involved in a complex endocrine regulatory mechanism of bone tissue growth and development, the paucity of catecholamines in AADCD may lead to anomalous skeletal development.

Although previous studies have not discovered significant MRI findings in AADCD patients, MRI is necessary for patients who present with neurodevelopmental delay to exclude other conditions in the differential diagnosis. Intriguingly, a study which focused on the relationship between MRI findings and AADCD illustrated that patients with AADCD usually have symptoms of abnormal brain development, such as cerebral atrophy, prominent subarachnoid spaces, and hypomyelination (Lee, Lin, Weng, & Peng, 2017). In the light of the results of this study and our patients abnormal MRI manifestations, which are consistent with each other, MRI appears to be a better prospect as an auxiliary diagnostic for AADCD, even though its sensitivity and specificity is open to debate.

AADCD patients are usually treated with pyridoxine/pyridoxal phosphate (PLP), monoamine oxidase inhibitors, and dopamine agonists as first‐line treatments. Gene therapy which has become an emerging approach for treating disease, was performed in a small group of AADCD patient with modest but promising results (Zwagerman and Richardson, 2012; Kojima et al., 2019). Patient 12, improved significantly on selegiline, a MAO inhibitors, while the other patients showed slightly increased limb muscle strength. It is reported that a 5‐year‐old female regular administration with a MAO‐B inhibitor improved her psychomotor functions (Kojima et al., 2016). Based on consensus guidelines, for patients without known L‐dopa binding‐site variants, L‐dopa trial can be considered if other treatment options fail (Wassenberg et al., 2017). Six index patients without L‐dopa binding‐site variants received L‐dopa (levodopa or Madopar), but the response was poor. However, patient 12 with L‐dopa binding‐site variants p.R160W significantly improved her psychomotor functions after L‐dopa treatment. Interestingly, the two patients with good treatment outcomes presented with milder disease than the others, and their motor development was much better than the others before treatment. Besides, the responses are often not satisfactory particularly in patients having severe symptoms. It suggested that the treatment response may depend, in large part, on the clinical phenotype.

Tay et al reported the cases of two Chinese AADCD siblings with compound heterozygous mutations IVS 6+4 A>T and 853 C>T presented with unusual mild phenotype (Tay et al., 2007); whereas Lee et al reported eight cases with homozygous or heterozygous IVS 6+4 A>T mutations, all showed severe phenotype (Lee et al., 2009). Remarkably, regardless of homozygous or heterozygous IVS 6+4 A>T mutations, all our patients presented with severe phenotype. The compound heterozygous c.1234C>T mutations were detected in 25% of the index patients, suggesting that the incidence of these mutations may be significantly higher in Mainland China. The current reported *DDC* variants includes 58 missenses, 6 frame‐shift, 9 splice‐site,1 in‐frame, 3 complex, and 2 nonsense variants (Himmelreich et al., 2019). Among them, approximately 10 missenses mutations associated with milder phenotypes. Patients may present with mild or moderate if found to be compound heterozygote or homozygote for one of the following missense or nonsense mutations: c.478C>G, c.853C>T, c.1123C>T, c.387G>A, c.665T>C. In our study, we discovered two novel missenses mutations, pD189Y and pC100S, which may also only cause milder phenotypes. It is important to recognize the milder phenotypes of the disease as these patients might respond well to therapy.

Patient 12 harbored the compound heterozygous missense mutation p.R160W and p.D189Y, and presented with moderate clinical phenotype. Interestingly, the patient described by Barth et al. harbored the compound heterozygous mutations pR160W and pR347Q and also showed moderate clinical features (Barth, Serre, & Hubert, 2012). Biochemical studies on the combination of two variants in heterozygosis were carried out only for the variants p.R347Q and p.R358H, and results revealed a positive interallelic complementation between the two variants (Montioli et al., 2018). We hypothesized that a particular combination of two AADC alleles, p.R160W and p.D189Y, may produce a phenotype that is different from the expected one, possibly due to interallelic complementation. Of course, in vitro expression systems should be used to investigate the enzyme kinetics and confirm the speculation, we will carry out dual‐vector expression systems to explore compound heterozygous genotype‐enzyme‐clinical phenotype correlation in the future.

## 5 CONCLUSION

The most intriguing aspects of our study are that we presented clinical details on a cohort of 17 patients in Mainland China with broad clinical variability, molecular spectrum, and different responses to medications. In addition, we explored *DDC* genotype with mild or moderate clinical phenotype correlation. In short, our research expands the clinical spectrum of AADCD and contributes to the knowledge of the genotype and phenotype correlation for the DDC gene.

## CONFLICT OF INTEREST

The author has no conflict of interest to declare.
